# Habit—Physiologically Considered

**Published:** 1853-07-01

**Authors:** John Addington Symonds

**Affiliations:** CONSULTING PHYSICIAN TO THE BRISTOL GENERAL HOSPITAL, ETC., ETC.


### Original Communications.

### HABIT—PHYSIOLOGICALLY CONSIDERED.

A LECTURE DELIVERED MAY 9th, 1853, AT THE BRISTOL LITERARY AND PHILOSOPHICAL INSTITUTION, BY JOHN ADDINGTON SYMONDS, M.D., CONSULTING PHYSICIAN TO THE BRISTOL GENERAL HOSPITAL, ETC., ETC.

I PROPOSE on this occasion to invite your attention to a class of phenomena, generally designated by the word Habit. This word, as you are aware, is one of various meanings; and yet through them all you will find a thread of connexion. It denotes that which has been observed so often in a living organism, that it is regarded as having something of permanency or constancy in a being which is evermore liable to change. It sometimes implies a cause; as when we notice that a friend has become stout or thin, and our suspicion is put down by the remark—"Nay, but he is of a full or spare habit,"—which means, that there is something in his original constitution, whether derived from inheritance or noted in an early period of his history, which is considered to be the cause of what has become the ordinary condition of this person's frame as to plumpness or leanness. Or it simply expresses a mode of action, the final or ultimate explanation of which is simply that it has occurred so often as to be always looked for. A stamp of permanence has been given to what at first might have been regarded as casual and temporary—that is, begotten of the circumstances under which it first originated, and to be expected to recur only under like conditions. This reference to habit as something ultimate or final, springs from the tendency in the human mind to acquiesce in the customary. From the unreasoning peasant, who quietly tells some traveller, startled by what he conceives to be a foolish or even outrageous mode of action, *that it is the custom,*—to the philosopher, who, unable to discover any more remote antecedent to the chain of sequences under his survey, says, that such is the natural order of events, the mental tendency is the same. A thing familiar is customary, and what is customary is acquiesced in. Nay, there is a tendency to imitate it passively. And what was an individual trick or habit, becomes a fashion or social convention.

Habit is sometimes used in a more vague signification, as when we speak of the habits of animals or their customary actions, and when it would be more correct to say, instinctive actions. But in this case, the habits are referred to as the effects of the instincts. And if the phrase "Instincts and Habits" of animals be used, it is intended to distinguish that mysterious principle called instinct, from the action which it prompts. Not, however, that habit in its strict sense may not be predicated of the lower animals; for as they are the subjects of volition and reasoning processes to a certain extent, so also they may be subject to habit.

It is not uncommon also, to speak of the habits of nations or races, meaning thereby, such modes of life and action as are usual to them. Perhaps in all the senses of the word, we shall find a connexion with its etymology, and that it bears reference to something which has been held (*habitus*), retained after being acquired; something added to the individual, and henceforth always associated with him. Again; *dress* is a *habit*, and *coutume* passes into *costume*. But we have to treat of habit in a more restricted sense, or as I have expressed it in the title to this lecture—"Habit physiologically considered." As physiology is the science which treats of vital actions, so we have to investigate habit with reference to these, or at least to some of them. We shall consider it in reference to those which constitute the life of relations in man—those functions which place him in relation with the outward world—sensation, motion, and thought. But while each of these heads will have some discussion, the greater part of our survey will be devoted to habit in relation to motion.

Some of the most curious examples of actions resulting from habit are to be found among those which we generally associate in our minds with the prosecution of some purpose, the relief of some feeling, the expression of some emotion, and yet in these cases there is no such final cause discernible. "Why does Mr. Thunderton speak so loudly and angrily? does the occasion call for it?" "Oh, that's his way—it is a habit he has got." "What does Mr. Scrutator find so very remarkable in us? Do you see how he fixes his eyes upon us?" "Oh! that's nothing—he always does so—it is his habit." "Don't you pity that poor gentleman? he is obliged to cough between every clause of his sentences." "Oh, no, he is quite well. We call it his interjectional or ejaculatory cough; and very expressive it is, like that of one of the fictitional characters of the day; it is a habit he has fallen into." "That person must surely be very unwell—very giddy.—See how he sways about in his walk—he will surely fall." "Oh! no,—he always walks in that way—he got the habit early in life." "What is that gentleman in search of? Do you see how he examines everything on the table and mantle piece? What is he looking for?" "That shows you don't know him! We never notice it. He has had that habit as long as we have known him, many years ago." These cases might be multiplied indefinitely. In each you observe that an action which appears to a stranger to be generated by a particular occasion or circumstance, is by one familiar with the subject of it explained, and seemingly quite to his satisfaction, as being an *habitual* eccentricity. That action, or manner, or mode of expression which strikes us as odd and exceptional, has in the observation of others occurred so frequently as to be considered necessary to the individual, the result of his acquired bodily or mental constitution. It is, in common phrase, "second nature,"—habit.

We are not, however, about to spend much time upon these abnormal instances. For in the category of Habit must be placed a great number of our most ordinary and familiar actions. Such are those actions which have become secondarily instinctive, or automatic;—those wonderful acquirements which we call standing, walking, running, grasping and handling, speech and talking, and those less universal but still not uncommon accomplishments of swimming, climbing, dancing—all the manual arts or handicrafts, and the fine or æsthetical arts;—all these belong to habit, as a principle of action, no less than do those actions which we call mechanical, and those aberrant movements which are designated nervous tricks and fidgets.

### Habit in Relation to Motion.

The function of muscles is, as you are aware, to move some part or the whole of the body, or to impress some motion on surrounding objects. A muscle is a bundle of fibres, which, in shortening themselves, bring their two ends nearer to each other. For various reasons, which we have not time to consider, several muscles act together in effecting particular movements. It is obvious, therefore, that they must act harmoniously, and to effect this they are under the governance of a central impulse. The muscular fibres are called into action by motor nerves, and the impulses in these several motor nerves are associated or derive a unity of origin from a nervous centre, as it is called, a part which receives an impression or imparts an impulse. Within the compass of the spinal cord and the brain, an immense number of these centres or ganglia are congregated, and so closely packed together as to seem to be one substance.

Some movements in the body are unaccompanied by sensation, and not instigated by the will;—these are often called the involuntary, and sometimes, instinctive movements. All the mechanism consists in the transmission of an impression by an afferent nerve to the ganglion which originates an impulse in the motor nerve. These movements may occur in an animal deprived of consciousness, or the susceptibility of feeling, as well as in one possessed of it. Thus, suppose the spine of a frog has been cut across the middle, if you pinch the extremity of one of the hinder limbs, the whole limb will be retracted. We are constantly the subjects of many such actions; one of the most striking examples is the series of muscular actions employed in breathing—a wonderful congeries of muscles which open the aperture of the windpipe, expand the chest, and so admit the fresh air, and afterwards expel the air which has yielded its oxygen to the wants of the blood; all these are put into action by afferent nerves, which convey an impression to the central respiratory ganglion, which sets in motion the requisite muscles. These movements are often accompanied by sensation, and may be altered, to a certain extent, by the will; but they are quite capable of going on, and in the healthy state they always do go on, without the one or the other, as we know at the present moment. Were other proof needed, it would be enough to observe the breathing in a person profoundly asleep or rendered insensible by disease, or in an animal deprived of those parts of the nervous system which minister to sensation and volition. This class of automatic movements has been called of late years reflex, or excito-motor.

There is another set of movements which are prompted by sensation, and are yet independent of the will. They are designated by some physiologists as consensual. Dr. Carpenter uses the term sensori-motor. I shall employ the term sensational; and, further, I think it desirable to discriminate these movements into two subdivisions—the subjectively sensational, and the objectively sensational. Now what do we mean by subjective sensations as distinguished from objective? An objective sensation is one that gives us information of something in the outward world, something that does not involve our consciousness or remind us of our own individual existence. I see a green meadow, or hear the song of a bird, or smell the fragrance of a flower, without my mind being cognizant of anything but the objects which have excited the sensations of vision, hearing, and smell. These, then, are objective sensations. The subjective are those in which the consciousness is involved, the *ego*, the individual self, partially or exclusively. I touch a cold marble: while I recognise the marble as the cause of my feeling, and attribute to it a certain quality which gives me the feeling, my consciousness, or an affection of me, myself, as the subject of the outward operation, forms a part of the sensation. But this is a mixed example, and might be called an objective-subjective sensation. Take another example:—I feel *ill.* I do not know what is the matter with me,—I have no pain. I am surrounded by pleasant objects, and within reach of everything likely to make me happy; but I feel ill. This is an intensely *subjective sensation.* Take one more example:—I have a headache, *i. e.*, I feel pain and I refer the pain to my head. This is a mixed sensation;—it is subjective so far as the feeling of pain implicates the ego; it is objective with reference to the seat of the pain; that is, the part of the outer world constituted by the body. It may seem strange to talk of a person's own body as being external to his conscious identity. But if you consider the matter fully, you will find that the knowledge of our bodily organs is an acquirement, and not a part of the feeling of personal consciousness. A young child suffering pain in some part of the body, is often quite incapable of saying where the pain is seated. In disturbed states of mind, the separateness of subject and object is very well shown with reference to these bodily feelings. I have known a person in delirium say to a bystander, "You have a dreadful headache, sir." *He* was the sufferer, but his morbid intellect linked the pain with the image of the person before him, instead of with his own body.

These sensations may then, I have said, whether subjective or objective, have motions immediately related to them; responding to them, as directly as those which are instigated by the will—often coincident with, and controllable by the will—but still having a causation of their own.

As an example of subjectively sensational motions I may adduce the act of coughing. A tickling sensation is felt in the wind-pipe; a series of muscular actions ensue, vehemently forcing the air out of the chest, and often not to be restrained or prevented by the will. Winking the eyes is another example. The movements which preserve the equilibrium of the body are mainly prompted and guided by subjective sensations, with but little and often no assistance from voluntary efforts. This governing feeling of equilibrium is directly related with pressure on the nerves of the feet, or whatever parts are supporting the rest of the body. If that compression be removed, giddiness, or a feeling of loss of balance is the result; and in an instant such muscles are thrown into action as tend to restore the equilibrium.

Of the objectively sensational movements, no instances are more striking than those which are associated with vision. Close one eye of an infant, and mark the movement of the eyeball as you shift the light before it—then let both be exposed, and see the admirable concert of action between them—and when you are acquainted with the muscles and their nerves that effect these movements, how nicely they are adjusted and balanced against each other, and with what delicacy they mutually respond, your admiration is great indeed. Though these actions are thus immediately related with vision, they may be, and very frequently are, the servants of our will, as when they fulfil our wish of looking in particular directions, or at different objects. But, in these cases, motion does not enter into our consciousness. The object of our desire is the sight of something. We *will* the seeing of it—that is, we look at it, inspect, examine it, &c., but we are unconscious that, in order to this, certain movements of the visual organ must take place. Here then is a palpable distinction between this class of movements and the voluntary: for in the latter, whether we know anything or not of the muscular instrumentality, we are conscious that our wish is to be executed by a movement either of the whole, or of a part of our frame.

It is in this group of sensational motions that our actions are most closely analogous to those which subserve the instincts of animals. They are as *blind*, that is, as dependent on motives involving no foresight, no prearrangement of our own. They are as perfect—for we neither learn them nor teach them—they are the works of an art unteachable, untaught—and they were as complete in our first parent, when his eyes feasted on the natural glories of Eden, as in his latest descendant, inspecting the wonders of art in the Crystal Palace.

Another class of movements are those which give outward expression to the emotions. These also occur without any intention or volition, whether they consist in the play of the features, or in the gestures of the limbs; joy, sorrow, anger, complacency, fear, courage, confidence, distrust, speak in the quick turns and ever-shifting hues of the eyes, in the varying surface of the cheek, in the expansile nostril, the flexile lip, and the smooth or knitted brow. To this head also belong those vocal movements which give utterance to particular feelings, and are common to all ages of the human being, and to all animals possessed of vocal organs. The scream of pain, the shriek of terror, the sigh of grief, the yell of resentment, the shout of joy, are the instantaneous productions of these passions. The emotions which approach nearer to intellectual conditions are also related with actions altogether involuntary. Such are the perceptions of the beautiful, the sublime, the wonderful, and the ludicrous.

These emotional movements are interesting in a point which has not received much attention. The feelings prompt the movements ; but it is no less true that the movements excite the feelings. And this, I think, is the key to some of those curious phenomena, often thrown into the vague category of sympathy. A sudden panic in a multitude, a burst of enthusiasm, will spread like lightning. The individuals look round and see countenances and gestures expressive of a particular emotion, of which, in the very process, they themselves become the subjects. But you may say this is nothing but the instinctive reading of nature's language of gestures and featural expression. The best instance is in those anomalous states of the nervous system, observed in persons subjected to mesmeric processes. Here the individual partially asleep, and with his will in abeyance, is at the mercy of any association suggested from without : close his fists and put his arms in a menacing attitude, and the emotions related with such movements are excited.* Passive imitation comprehends movements very analogous. Yawning is the expression of weariness. The sight of yawning engenders the related feeling, and our own muscles answer to it.

Our next class of movements would be those which are directly prompted (still without volition) by ideas; meaning by this term, remembered sensations and thoughts, as distinguished from those objects of consciousness which are immediately brought from the outer world. But as the best illustrations are derived from the group of allied movements which we shall have to consider as pertaining more particularly to the topics of this lecture—that is, the secondarily ideagenous—I shall not now adduce them. I must content myself with remarking, that in various kinds of passive imitation we have specimens both of sensational and of ideagenous movements. You know how readily some persons contract a particular tone of voice from association with others. Now as the individuals often wish to avoid the infection, and yet do not escape it, the imitation is clearly passive and mechanical—the vocal movements which give the particular tone respond instinctively to the sound which has been so often impressed on the ear. But take a case of unintentional mimicry : you must have observed that when any one is relating an anecdote respecting the sayings or doings of an individual who has marked personal peculiarities, the narrator's face, tones of voice, and gestures, will represent the individual, though the operator has no intention of so taking him off (as the phrase goes). The idea of the individual is so vividly before him,

* The explanation of some of those strange mesmeric phenomena is admirably set forth in Dr. Carpenter's "Human Physiology." The chapter on the Physiology of the New System in the last edition, abounds in views highly original, and stated and illustrated with wonderful perspicuity and affluence. The members of this Institution should be proud of this production of one to whose voice these walls have so often echoed.

that he cannot help this imitation; and consequently his vocal muscles, those of featural expression, and those which govern the gestures, the gait, carriage, and demeanour—all obey the mental impression, without the intervention of any volitional action.

Let us now inquire into the distinction between automatic and volitional action. We have seen that in the several groups comprehended under the former, motion succeeds to impressions, without sensation or with sensation, to emotions, and to ideas; but that there is no indication in any of these instances of the self, the conscious ego, being the originator of the movements. The common statement is, that we will the action of certain muscles or groups of muscles, in order to execute our purposes. But this is an incorrect statement; for it is obvious that in childhood, and indeed at all ages, unless we have learned anatomy, we know nothing about the mechanism whereby we move our limbs. Nay, after we have acquired such knowledge, we cannot make use of it in the manner supposed. I cannot, by an effort of my will, cause the contraction of that muscle which is the chief agent in bending the elbow—yet that muscle may, in morbid spasm, be thrown into single and separate action. But, will the bending of the arm, and it is done. In many of what we call voluntary movements, we do not even will the action of particular limbs. Thus, in learning to walk, the infant has no notion of limbs, of planting the foot on the ground, keeping one leg stiff, rotating the body on the hip-joint, &c. It only wishes to move to some place or person in view. The motions ensue upon the desire, guided by the subjective sensations of equilibrium, and by the objective sensations of vision.

The more we consider the subject, the more plain will it appear that the exercise of volition consists in maintaining an association between certain ideas, sensations, and motions; the individual self being conscious of its relation as the cause of those actions, and of its power to increase, or lessen, or interrupt, or arrest them. If we wish to analyse the process in any given case, we must be careful, for reasons which will soon appear, to select one that is not an habitual or mechanical action. In learning to play upon a musical instrument, there are many movements to be acquired which are quite new, and would, perhaps, never be required at any other conjuncture in our lives. Or to take a less uncommon case. You have to lift the third finger alone, while the others are kept flat on the keys of the piano. The master tells you to practise it,—and what do you do in practising it? You keep your mind on the end in view,—the raising this finger so reluctant to act alone, vehemently desiring to be successful, and sooner or later, if you are free from any physical defect, your ambition is realized. This little achievement seems a simple affair, but it is really rather a complex one. It is not the mere lifting of the finger, it is keeping the other two down; for the finger in question is raised by a muscle with tripartite tendon, of which two divisions go to the second and third fingers, and therefore the tendency is for all three to move together when the muscle is set in action. To prevent this, the muscles that place the first two fingers are put into action so as to antagonize the extensor and keep them down, while the action of that extensor is allowed to tell on the third only.

But of all this the successful young lady is unconscious. She has only, by diligent application of her mind, kept the object steadily in view, and a wonderful association of muscles has done her bidding, she is unconscious how; she knows no more of the process than if she had called to her aid some of the fairies or genii of her nursery fables.

The process in volitional movement is well illustrated by what takes place in imitating an instinctive action—as in acting or forcing a cough. We conceive the idea of the act of coughing with the will of producing the act; and this follows with more or less success according to the vividness of the conception, and the readiness with which in the individual ideagenous motions follow the idea. In one who has histrionic genius, there is, besides the higher mental faculties, a quickness of response in the muscles to the ideas in the mind, as well as to the emotions.

But perhaps this subject may be further elucidated by considering what takes place in volitional efforts of thought. Much of our thinking is done mechanically—automatically—one thought suggesting another by the laws of casual association. But what is implied in such phrases as mental effort, application, undivided attention, painful abstraction of thought? There is a close analogy between this process and muscular exertion. They are analogous in the circumstance that they for the time engross our consciousness, that we feel a strain, an exertion, which, but for some ulterior object, we should be inclined to relax, and that, after it is over, we may be conscious of fatigue or exhaustion.

If you watch a child who, under the stimulus of emulation, or the fear of punishment, has been laboriously conning a task but just within the reach of its faculties, you may mark in the countenance, the skin, the pulse, the whole demeanour, effects like those produced by excess of bodily exercise. In both cases there has been an undue consumption of force. Wherein consists this mental labour? It is the forcible bringing together and keeping together ideas or signs of ideas, which have no natural tendency to cohere, or are not helped to cohere, by any other process than volitional effort. Take the instance of a child's committing to memory a rule of grammar, the meaning of which is altogether dark to him. I remember one, who before he was five years old, sat down to learn off the Latin Accidence (prompted by ambition—an elder sister having just been initiated into its mysteries), and he worked away at that luminous sentence—"A noun is the name of whatsoever thing or being we see or discourse of." Plain as this seems to us, it was to him only a series of words which had to be impressed on his mind, and remembered in the order in which they followed each other. And how did he achieve this wonderful acquirement? He read them over and over again—sometimes in silence—and then those cabalistic verbal signs were associated as visual remembrances. He read them aloud, and certain tones were associated with them,—reproductive of each other. The words contracted relationship, not only with the part of the page on which they stood, but with the part of the room where he sat, with the furniture near him, and with the emotions of which he was the subject, as hope and fear fluctuated in his anxious ambitious bosom.

At last the lesson was learned; that is, the words would come back into the mind in the series in which they had been seen in the book. They were no longer held together by mere sight and volitional attention, but the occurrence of the first brought the second, and the second the third, because they had now very often co-existed, or proximally succeeded each other; and they had come to be associated with the images of other visual things, the recurrence of which was easy, by virtue of the liveliness of the original impressions.

To *attend*, or perform the mental act of attention, is to keep before the consciousness, by an effort of the will, particular impressions or trains of thought, or muscular actions. As to sensation, impressions excite the consciousness, and become sensations when the consciousness is not too closely associated with some other impressions or thoughts, or when they are new or very vivid. It seems to be a law, that consciousness does not link itself with any impression frequently repeated, unless it be accompanied by a feeling of pleasure or pain, or associated with some strong emotion, or with a train of thought associated with such emotion, (in common language, some interesting subject). The first time you sit down in a room close to a railway, and hear the roaring of a train, you think that it will arrest your attention every time it occurs, but when the novelty has ceased, you cease to hear it. It is not associated with any emotion or interesting series of thought; it is the passing of a train and nothing more. Or suppose you are at a dinner party. There is an incessant clatter of knives and forks and changing dishes, tramp of servants, and hubbub of voices, and yet you hear nothing but the words of your friend beside you, who is engaging you in some interesting dialogue. The consciousness at that time coheres to your friend's speech, fastened by the attractive force of present emotions, and is not to be torn from it by mere sensory impressions, unless these come reinforced by the divellent affinities of stronger emotions, or unless a voice pitched in a higher key than the confused Babel around you compels attention by its novelty. But the laborious action of the will in attention consists in enforcing a coherence between the consciousness and certain objects which are not bound to it by emotional interest.

But if there be labour or difficulty in this volitional effort, there is still more in that which has for its object the conjunction of past sensations or ideas, to the exclusion of the objects of sense. Suppose a young person shut up in a room and set to write down his notion of the character of Alexander of Macedon, and suppose, if it be possible, that the youth has never taken any interest in that magnificent hero—to measure the efforts he has to make you must consider what objects would be most likely to present themselves to his consciousness were he not to endeavour to control his thoughts. The things in the room, furniture, prints, &c., or should there be nothing of interest within, then the view from the window;—escaped from these temptations he has to resist the attractions of memory, some sport, or entertainment, or curious book, or some prospective pleasure. He has to keep before his consciousness the image of Alexander, and all the cluster of actions and sayings associated with that idea through books, lectures, &c. No difficult task; but he has to arrange and parcel these recollections in connexion with certain qualities of Alexander's mind, and clothe them in appropriate language. These connexions are not so spontaneous as the others, and require more volitional effort.

The effort in *recollection* consists in detaining before the consciousness some idea about which the wished for ideas will cluster by association. We cannot *will* their reproduction immediately, for, were they objects of volition, they would already be in the presence of the mind, and therefore need no summons. Do I wish to remember the name of some person whose image is in my mind's eye? I keep it there—my thoughts about him take the forms of place and time—each of these bringing a large cluster of associations—to some one or more of which the *name* so adheres that it at last is presented to my consciousness.

There is a great difference in the facility with which ideas are presented to the consciousness and that by which they are detained. Conjunction between ideas and the consciousness, is greatly *assisted by the emotions.* An illustration will bring this before you immediately. The first time you gathered a violet you were in company with a friend. Long years afterwards you come into a room perfumed with violets which you do not see. The fragrance recalls the flower, its size, colour, form, &c. Those perceptions coexisted in the first instance. The presence of one has recalled the rest by the mere law of coexistence. The same law brings before your mind at the same time the friend who was with you; but he is associated with so many emotions that his image remains long after the violets and their perfume have faded from your sense and memory. Nay, you are so occupied with those remembrances that you may fall into a fit of abstraction, that is, be unconscious of anything but the ideas and sentiments which have thus been summoned to your consciousness. Thus you see the ideas and remembrances associated with emotions are not only before the consciousness, but are detained there; while those remembered sensations which had no other connexion than that of coexistence and succession, passed away as soon as they appeared.

These remarks on attention will enable us to return to volitional motion. The will compels a conjunction between the idea of the movement to be executed and our consciousness. To the idea of the movement, conjoined with the wish to execute it, the nervo-muscular apparatus responds sooner or later, if it be within the compass of our organization. The action is much aided by the senses, and, in some cases, particularly by the muscular-sense. Indeed there is no voluntary action of which sensation does not form a link in the series of events. This is well seen in the act of prehension. In grasping a ball for the first time, my fingers obey my wish to close upon it. The degree of force with which they will close depends on the sensation which the ball gives to the nerves of touch, and the feeling of resistance afforded by the muscular sense,—that is, the nerves of sensation in the muscles. To hold the ball in my hand, I must either keep my attention fixed on the action, or the nerves of sensation must maintain the muscular action. If these nerves be paralysed, the action must be guided by the attention and by sight. The following case, related by Sir Charles Bell, is a good example:—

"A mother, while nursing her infant, was seized with a paralysis, attended with the loss of muscular power on one side of her body, and the loss of sensibility on the other. The surprising and, indeed, the alarming circumstance here was, that she could hold her child to her bosom with the arm which retained muscular power, only so long as she looked at the infant. If surrounding objects withdrew her attention from the state of her arm, the flexor muscles gradually relaxed, and the child was in danger of falling."—*Bell on the Hand*, p. 244.

These consensual changes have something secondarily automatic or reflex in their character, for although when I fix my attention on the action, I am conscious of a sensation; yet when I am occupied with other subjects, as in talking, I have not the sensations, and yet the nerves of sensation are acting in concert with the nerves of motion, and maintaining the action. The sensational has been converted into a *reflex* action.

Next we may notice the connexion between volitional action and sensation in the process of speaking or singing. We need not go back to babyhood, though the study of it is most interesting; but we will tax our mature consciousness for the supply of information on this subject. I am learning a new language. I wish to acquire the pronunciation of a new sound—say a German guttural. It is a volitional effort. I hear the sound—I fix my consciousness upon it; my vocal muscles, after a few trials, produce it. After a time no effort of volition is needful. Certain letters associated with the first hearing of the sound suggest the idea of the sound, and this the muscular action. The action of the muscles of the voice clearly, then, belongs to the sensational group.

Strictly volitional movements are those which are objects of mental attention, combined with the wish to execute them, and which are not performed but under such circumstances. They are thus distinguished from actions which, though coinciding with, and controllable by our will, are originated and maintained by processes independent of this mental principle.

We have now to inquire how these volitional movements, after a time, pass into the category of instinctive or automatic actions. In fact we are entering the domain of Habit—a field which, though appropriated especially to this discourse, we have had to approach by a circuitous and, I fear, tedious avenue.

*Habitual* motions are those which have been transmuted from volitional to instinctive,—which have become secondarily automatic,—which from having been compounded of will, idea, and sensation, have become merely sensational, and perhaps, even in some cases, purely reflex. The ego—the consciousness, which was the first mover, has been able to leave the transaction to its subordinate agents, while it is occupied with other actions, or with sensations and thoughts requiring its undivided attention. Of these many have been established in early life. In standing and walking we have examples of complicated series of muscular actions guided by the sensation of equilibrium, and becoming ultimately all but reflex, though originally prompted by the will. That the will is originally concerned we see, not only by our observation of children learning to stand or walk, but also in adults in whom the apparatus has been weakened by illness or old age, and in whom the mechanism is no longer so self-acting as not to require that mental attention to the several stages of the process, in which volitional action consists.

*Speech* is another of the habitual or secondarily automatic actions. In this process there is the perception of sound as connected with some object of sight (as in the naming of a thing) and the wish to imitate the sound. The action of the vocal muscles is preceded by sensation, idea, and volition. But after the habit of speaking has been acquired, it becomes purely sensational or ideagenous without intervening volition, and is allied to the instincts.

I here take the liberty of quoting from a paper which I published many years ago:—

"The articulation of every word was once, perhaps, the result of effort, a voluntary exertion of the vocal organ to imitate a sound produced by another. But now it is enough for the word to occur to the mind, and the pronunciation follows, without any intermediate volition, merely because the idea and the action have been accustomed to the relation of antecedence and consequence.

"Again: I may use some word which I not only did not intend, but which I would much rather have avoided, as it may be personally offensive to the individual with whom I am conversing. This word, in all probability, will be found to be similar in sound to that which was present in my mind, but which was not expressed by my voice. The word was the product of a certain aggregation or series of vocal movements, which followed some initial movement common to it, and to that other series which properly belonged to the idea in the mind. This we conceive to be the meaning of what is commonly called a *lapsus linguæ*, and is very different from a malapoprism: *the latter is a mistake of the mind, the former is a mistake of the muscles.* A similar error not unfrequently occurs in writing. A perfect master of orthography may commit a mistake of this kind; he may write, for instance, the adverb *there*, though the pronoun was in his mind, merely from an irregularity of muscular succession. The tracing of a word on paper is the result of a particular set of muscular movements; but words of very different meanings may have very similar sets, and even initially identical, as in the instance just mentioned; and hence the mistake arises. We have heard persons say that a bad pen would make them mis-spell; in such a case, the impediment offered by the pen causes an irregularity in the succession of the movements. But it may be asked, how is it that we sometimes utter or write a word no less dissimilar in sound and in symbolical characters, than foreign to the subject discoursed of? The causation in this case is different; the error exists in the mind, and arises from our being occupied with more than one series of ideas; in which case an accidental exchange takes place between the series communicated and that which is retained. To a person engaged in writing when others are talking around him, the accident is very likely to happen. Some word makes a particular impression on his mind and diverts him a moment from his previous train of thought; but his muscles continue to act, and follow the impulse of the word in question, as of any other that passes through his mind, and germane to the matter in hand.

"From what has been said, then, it is deducible that there are motions immediately consequent on ideas, in the same manner as others consequent on sensations and emotions; but we have not arranged the former in a separate class, because we are not aware of any evidence that *ideas* assume the relation of proximate causes to *motions*, except under the operation of the general law or principle which we have been engaged in illustrating, while sensations and emotions, on the contrary, manifestly produce their appropriate actions, without any reference whatever either to previous association or succession."—*Relations of Mind and Muscle. West of England Journal*, 1835.

Such actions as standing, walking, speaking and handling, are the most primitive arts of life, belonging to man as a species, and contemplated as essential parts of his active existence, without which, indeed, he would be wanting in the outward characteristics of humanity. They correspond to actions which in the lower animals are all but coeval with birth, or which only require the complete development of the organism rather than any process of education. The young kid walks on the day of its birth. The bird does not fly when just out of its shell, because its wings are imperfect.

In these primitive arts, then, belonging to the whole race, there is a more ready and rapid conversion of volition into habit or instinct than in others, which I now proceed to notice. These are the arts acquired by long education and practice, and which belong either to individual man originating them, or to individual man affected by his fellows. They are the manual, the domestic, the social, and the fine arts. In all these arts the limbs have to respond to and work out an idea; and the connexion must be frequently established and repeated by volitional efforts before the art becomes a habit. And yet we shall see here, again, the power of habit, and how, when it is more early acquired, it becomes allied to an instinct or an inspiration.

It is in these arts, indeed, that we may discover some of the most marked examples of habit,—recurring to that character of the principle which we gave at the onset of this discourse, as the conversion of the casual or temporary into the fixed or permanent. For as the muscular actions have been engendered to meet circumstances which are not necessary to the life and endowments of the species; in other words, to subserve wants and answer to ideas which have arisen under particular circumstances or in particular minds, so the conversion of them into actions that may occur without the maintenance of volitional supervision, illustrates very strongly the dominion of habit.

Such combinations of muscular actions as are effected in the sleight-of-hand tricks of a juggler, or in the scarcely less surprising dexterities of an accomplished player on the violin, may never before have transpired, and may never again transpire in any son of Adam, and yet in that individual they may have been so frequently produced, (*i. e.*, the will may have so often effected a junction between the idea, the sensations, and the motions) that the succession is maintained without any mental attention. The player may talk to you while his hand is bringing out the music; one note suggests the next which has so often proximally succeeded to it, and with it comes the nervo-muscular action according to the principle of association which we have before adverted to. But in cases of this kind we may sometimes in our analysis fail to discover any link formed of an idea or a remembered sensation. Thus, one may begin to whistle without the faintest notion of what tune the muscles of the mouth and cheek will modulate. The muscular actions aggregate themselves into combinations and series unprompted by an idea, unguided by the will, nay, unsuggested by a sensation (for the motion precedes the note evolved), and arranged solely by the law of previous coexistence and proximal succession, repeated an adequate number of times. They are mechanical, automatic, reflex. Originally, perhaps, the order of events had been remembered;—musical sounds associated with certain words. The recurrence of the words brought the sounds, to which the vocal movement responded. But now the first movement sends an impression to the reflex centres, and these maintain the series independently of the sensational, ideagenous, and volitional centres. It is like the running off of a musical machine, except that the series may be deranged at any moment by an idea or emotion or sensation. Therefore it is most likely to be safe from interruption when the mind is in that blank condition to which the most sentient, sentimental, and intellectual are sometimes subject. Therefore there is a physiological meaning in the old line:—

"He whistled as he went for want of thought."

The habitual or *secondarily* automatic actions bear resemblance to the *primarily* automatic in the circumstance that when once established they are liable to derangement rather than assisted by mental attention. Fix your mind on the process of breathing, and it becomes laborious or irregular,—think of the act of swallowing while you are performing it, as in taking a pill, and the action becomes spasmodic, convulsive, abortive. In like manner if you attend to your walking as you pass from one side of a drawing-room to another, it is ten to one but that your gait and carriage will be a series of jerking, swaying, rolling motions, in short, awkward, constrained, ungraceful. Something in such cases must be set down to emotional causes, such as the disturbing influence of anxiety. But from what we remarked before, you may be prepared to admit that the act of volitional attention is closely allied to emotional processes. And it will be found that in certain of the secondarily automatic *mental* operations, this disturbing influence is still more easily traceable.

The readiness with which these habitual movements are established I have already mentioned incidentally, is closely allied to natural aptitude or talent. It is far beyond dispute, that however great may be the power of education and practice, yet that men differ immensely from each other in original capability for particular arts. Men are *born* artisans and artists, as well as poets; for the former, whatever may be the mental requirements, there must, at all events, be an aptness of hand, a quickness of response in the muscles to the ideas in the mind—a readiness for the establishment of those series, and aggregations of complex co-ordinate movements, which, for their first institution require the repetition of volitional efforts, but which in one man require almost an indefinite number of repetitions, while in another a very few will suffice to convert them into habits. This, however, (natural dexterity) is but the bare mechanical substratum of skill in art. Yet without it a man cannot excel, though his mental capability for the higher processes in the art be of transcendent excellence. He may have the most nimble apprehension, a memory the most retentive and accessible, the most refined and delicate sense of the beautiful, a power of rapidly combining and abstracting those qualities in natural objects, which, when reproduced, will faithfully and vividly represent the originals,—an instinctive knowledge of that subtle symbolism whereby objects are made to evoke in other minds those sentiments which were inspired in the artist as *he* looked on nature, and with all this, a noble desire to make the æsthetical gratification minister to its natural handmaids, Virtue and Religion; he may have all these, and many more qualities, necessary for the accomplished artist, and yet they may only serve him for the appreciation of the achievements of others,—not because he wants "the vision and the faculty divine," but because the hand is not a defty servant of his thought. But when, indeed, the hand and the eye and the mind do come together, each with the highest qualities, compass, quickness, and co-ordination, then we have a Phidias, a Michael Angelo, a Raffaelle. Many like instances might be adduced; but I hasten to remark, in connexion with this department of our subject, what indeed is well known to every one, that for the formation of many of those habitual co-ordinate actions, nothing is needed but frequency of repetition. They argue no special fitness of organization (though, indeed, even in them a natural cleverness may be discernible), and merely instance the power of repetition. Such are the mechanical processes of every-day life,—the act of writing, the operations of the toilet, the dinner table, &c.

But here the question naturally occurs, why should mere repetition dispense with volitional exertion? To answer this fully, would require a long and abstruse discussion. I must content myself with a summary statement of what seems to me to be the explanation of the fact. The more the action of an organ is augmented the stronger it becomes, and its nutrition increases. This is clearly seen in the development of muscles called frequently into play. I would apply this fact by analogy to the nervous centres. The different nervous centres in the encephalon communicate with each other by commissural fibres. In volitional movements, the action of the commissures between the sensational or ideagenous centres and their related motor centres is excited in the first instance by the will. But the repetition of more or fewer of these incitements appears to suffice for the growth and development of the commissure to a degree of strength and activity adequate to the performance of its functions independently of the will. Thus the volitional action is converted into a habit.

The anormal habits are curious, and perhaps less easy of analysis. They are those actions which subserve no apparent purpose, either as to the functions of the whole system, or to the will, or the emotions, or the ideas of the individual. What earthly end is answered by biting the nails, twitching the hair, twirling the thumbs, rubbing the hands, kicking the heels, and various other singular and all but unaccountable human actions, which are designated in the vernacular as tricks or fidgets? Why should a person under strong emotion, exhibit such peculiarities of deportment as are attributed to Sir Jacob Kilmansegg on the morning of his daughter's christening?

> "And Sir Jacob, the father, strutted and bowed,
> And smiled to himself and laughed aloud,
> To think of his heiress and daughter—
> And then in his pockets he made a grope,
> And then in the fulness of joy and hope,
> Seem'd washing his hands with invisible soap
> In imperceptible water."

Why was it that when a certain great statesman in the House of Commons was about to make a signal rhetorical effort, his friends were advertized of it by his mechanically rattling his watch-chain and seals for full half-an-hour before he rose? Why do little boys, in saying their lessons, go through sundry mysterious operations of buttoning and unbuttoning their jackets; and why do gentlemen, earnest in argumentation, wildly lay hands on the little implements of a neighbouring lady's work-table, and turn them to strange uses on the adjoining furniture? There must be a law for facts so common, and all but universal.

I believe that they are referable to two principles—one of them is the provision made in the nervous system for the concurrence of muscular action with deep thought and strong emotion. Consider how nearly all the emotions have their natural alliance (in the form of expression) with certain muscular actions in the features and the limbs, and how very important these are in the mutual communication of men in early states of society. Consider also, how often some form of muscular action goes with thought; and again, how common it is to accompany speech with gesticulation; and how in the more important acts of life, thought and motion are going on together. In literary composition, the action of writing is a natural accompaniment. If a man dictates, it is ten to one but he will walk about the room while doing so, because it is natural for some muscular action to accompany profound thought.

But you may say that these considerations do not help us out of the difficulty. For speech and gesticulation being the accompaniments of thought and emotion, why should we add such unmeaning muscular actions as those which have been adverted to? We come then to the other principle, which is, that the motor centres are apt to elaborate more force than is required, and which must be disposed of in some way. Now that there is such a thing as nerve-force cannot be doubted. We talk of force of attraction, of chemical force, of motive force, of electric force; and though none of these forces can be produced as entities, and may be only summary expressions of certain chains of phenomena considered in their causative order, there is as much reason for using the term nerve-force as any of the others. It is that principle or power which, generated in nervous tissue by virtue of the changes which this substance undergoes in its ultimate muscular nutrition, (in the higher animals, under the operation of blood-changes,) stands in the relation of proximate cause to motion, sensation, and the material ministrations of thought. The common sense or consciousness of mankind, attains to a very near recognition of this power, when it assigns the buoyant movements of childhood to an excess of vitality, and when a man says that he feels such a surplus of irritability or irritation in his system, that he takes active exercise to get rid of it. We have seen how the nervous centres are brought into action simultaneously and co-ordinately. Now if there be great excitement in one, (excitement means exalted vital action,) there will, to a certain extent, be the same in others, more especially in those related. If the emotional centres are strongly excited, there will be a large elaboration of force in the motor centres, some of which force may be discharged in the appropriate featural expressions and gestures; but to expend the whole of it, strange movements and antics will very probably be performed. To let these have free play is a great relief to many persons, while to restrain them requires great self-command and discipline.

In sleep, although the centres of sensation are quiescent, there is often too much activity in the motional centres, and then the nerve-force goes off in a variety of irregular muscular actions, such as kicking, stretching, change of position, tossing off the bed-clothes, &c.

In sensation, in motion, and in thought, nerve-force is generated and consumed. The fatigue, nay the absolute bodily exhaustion, induced by excess in any of these functions is a proof of it. In persons of the nervous temperament, there is at all times an amount of nervous energy produced beyond what is absolutely required for the performance of the normal functions of the brain. Hence it is in these persons that we see the most striking instances of the tricks which we are now considering. Very remarkable is the tendency in some children. They wink their eyes, make strange contortions in their limbs, change their posture, shift the weight of the body from one foot to the other incessantly when answering the slightest question, and all from the excess of motor nerve-force generated concurrently with the little mental excitement. These movements sometimes occur in particular sets of muscles in preference to others, and then they are called habits.

But I cannot leave this part of the subject without remarking that there is good reason for the caution which has often been given, not to interfere too much with these nervous habits; for they are sometimes salutary outlets for morbid irritation. I know a young lady who, when her mind was unusually interested, had a habit of rubbing her forehead, and if this were prevented, either by moral or mechanical means, it seldom failed to induce threatenings of serious disorder in the brain. There are certain kinds of convulsive seizures which may be considered as discharges of nerve-force, like those of electric jars, highly charged. Pardon me for giving you one more practical instance: There are some persons whose brains, when highly excited, say, by a public entertainment, or an interesting conversation or argument, or even by having fallen into some engrossing train of thought, cannot pass readily into the normal condition. The elaboration of nerve-force continues. If they retire to rest they cannot sleep; they go on thinking, and feeling, and tossing about, or vividly dreaming, when they ought to be "mere clods of the valley." Now the best thing to prevent this, is to take active muscular exercise before retiring, whereby the superabundant force is used up.

### Habit, as to Sensation.

On first considering the effects of habit in connexion with sensation, it would seem to be the reverse of what occurs in connexion with motion. Habit, instead of increasing, seems to lessen sensation. We speak of becoming too habituated to things to feel them. The contact of clothes with our skin is habitually disregarded, though, had the investiture been a new ceremony, we should not be able to forget them for an instant during the day. We sit in a room reading or talking, while a time-piece, to the sound of which we are accustomed, strikes the hours and the quarters, without hearing it. The inhabitants of miserable tenements in the worst parts of our towns, (where sanitary matters are neglected to a degree that is a disgrace to civilization, and a dishonour to Christianity,) live unconscious of odours, which strike those who have not been inured to this wretchedness with disgust and terror. The "common light of day" gives us no subjective sensation, no feeling in our eyes, unless we have been unusually excluded from it for a long time, and then it may be blinding.

After considering such facts as these, we remember on the other hand, that education is known to quicken the senses, that the North American Indian is said by habit to see and hear sights and sounds which escape his more civilized brethren; and that the purchaser of tea can try forty or fifty sample-cups in succession, and make his selection of the best flavours with unerring accuracy; and that wine-tasters can, by practice, attain to the delicacy of discrimination which characterized those notable rivals vouched for by Sancho Panza, of whom, as you remember, one declared that the wine tasted of iron, and the other of leather, and each of whom was found to be correct when the cask had been drained to the bottom: for there lay an old cellar-key, with a thong attached to it.

These two sets of facts seem at first sight to contradict each other, but they do not really do so. We have seen that habit in reference to motion strengthens and gives independent power to a connexion established in the first instance by volition. It will be found to be the same in sensation.

What is the process in sensation? An impression is made on a sentient nerve, and conveyed by it to the sensory ganglia, where it becomes connected with the Ego, and is then, and not till then, a sensation.

We have already been speaking of attention, and shown that impressions are presented to the consciousness by virtue of their novelty or vividness, and are detained or dismissed according as they are associated or not with emotions, or compulsorily associated by the will. It appears to be a law that a repeated impression ceases to become a sensation when its novelty ceases. A different degree of vividness is in fact a new impression, and therefore comes under the law. The final cause of this arrangement is obvious. Were we at the mercy of all the impressions which are perpetually made upon us, were they all converted into sensations, connected thought would be impossible. But the impression may have nothing of novelty, and yet its recurrence will always be followed by sensation, because it has been associated with an emotion. This effect of association is strikingly exemplified in sleep. Impressions of the most violent kind may be made, and yet the sleeper will not feel them if he has been accustomed to them, and not had them connected in his mind with some subject of interest. It is told by Dr. Carpenter, of a great naval officer, that he could sleep through the loudest noises on ship-board, but if any one whispered in his ear the word *signal*, he was awake in an instant. So I know the case of a lady who sleeps profoundly during ordinary noises, but if her relative who is near her, makes in breathing a sound which she has often known to be the precursor of an attack of illness, she wakes immediately. So also with the tick of an alarum.

In the sharpening of the senses by education and training, we again see the influence of the emotions, or of ideas associated with emotions. We look and listen, that is, we attend to sights and sounds, because there is an expectation of good to be received, or a fear of evil to be averted. Again we are said to acquire the habit of attending to certain classes of sensations. Here the emotional interest is more remote. The objects of sensation may be uninteresting, but we exert our volition to compel a coherence between the impressions and the consciousness; and the conviction having been often repeated, and so once established, becomes permanent; the volitional becomes automatic. Habit supplants the will; and thus we have another illustration that the influence of this principle is exerted by virtue of association.

Habit would thus seem the offspring and afterwards the successor and representative of volition. But what is meant by a habit of inattention, a listless habit? Thus a person may fall into the habit of sitting at church and not listening to the service or the sermon. The use of the word in this manner is a metonymy. It is not the habit of not attending, but the habit of attending to your own thoughts in preference to those of the clergyman.

Everywhere in this subject we fall back on the principle of association. Everything in this world is interesting, or important, or influential, according as it is related with other things.

The repetition of volitional impulses gives increased strength to the related centres of motion, because these centres become active. The repetition of impressions which are not taken up by the emotions or the will, and do not pass into sensations, will come to nothing. It is not that habit deadens the sensibility to the repeated impressions, but that the impressions ceasing to be linked with the emotions and the will, having in fact no associations with any thing else in the mental life, die away; or, indeed, never arrive at any thing but an abortive existence.

### Habit as to Thouht.

In the remarks preliminary to our consideration of volitional motion, I spoke of some of those mental processes which are enforced by the will in efforts of memory. There are others to which I can but very briefly advert, with a view to the prosecution of our immediate subject. Such is the compulsory contemplation of phenomena in a certain order or arrangement, when we reason, whether inductively or deductively, or when we arrange, according to a certain shape or plan those clusters of ideas and emotions which belong to fancy and imagination.

Congeries of ideas, or trains of thought, originally associated or marshalled by volition, may, like the volitional movements, become automatic. The memory of compositions, or of narratives, or of arguments, once attained by efforts of the will, becomes automatic. Recollection passes into remembrance. Nay, like the analogous movements, it is apt to be deranged by mental attention. How often does a person find that the more he tries to remember something, the more he is baffled. He gives it up, talks of something else, and that which had been sought for in vain, comes unbidden. The automatic process of association went on better when the consciousness was not fixed upon it.

Arithmetical calculations illustrate this principle. That 2 and 3 make 5, is at first committed to the memory by means of the will,—after a few repetitions of these three figures, the combination of the two first will bring the third mechanically, that is, 3 will add itself to 2, and bring 5 in its train, and so on with a long column of figures. Mr. Dugald Stewart adduces the case of an expert accountant running his eyes up a column of figures and arriving at the sum total, unconscious of the intermediate mental acts, as an instance of a succession of efforts of attention, so rapid as to have been forgotten. But there seems to me no need of presuming that of which there is no proof. The numbers seen have so often been linked with their equivalents in the memory, that the latter recur passively and mechanically. Thus in consequence of long use and practice 2 and 3 cannot but suggest the idea 5, and 5 meeting 7 brings 12, and 12 meeting 6 brings 18, and with 18 added to 9, 27 links itself, and so on.

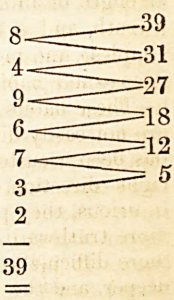

Thus the visual and the mental trains of figures interchange without any effort.

These associations have so often been compelled, that they now recur by necessity. It is just as with the player on an instrument; the impulses in the centre of motion so often associated with the notes of music on the paper come without attention, though at one time their co-ordination was only effected by dint of the most laborious attention.

In reasoning, a like automatic process is traceable. The *ergo* fastens itself without effort to the premisses. The conclusion is self-evolved from the major premiss.

And the self-evolution of thought may be traced much further.—Ideas grow out of ideas, without our consciousness of the various stages of their mysterious genesis. The new production suddenly blossoms forth to the astonishment of its possessor, who finds himself all but unawares the maker of a grand discovery. Or to vary the illustration, take the case of a writer of works of imagination. His mind has been stored with a vast assemblage of images, scenes, sentiments, and characters. In some auspicious moment, and sometimes unexpectedly, the scheme of a poem or fable unfolds itself before his mental eye; it is as if his mind up to that time had held all the scenes, incidents, characters and similies in a state of solution, and then by a happy conjunction of outward and inward agencies, the several elements of thought attach themselves to each other by their several elective affinities, and crystallize into their appropriate shapes and colours of beauty and grandeur.*

The secondarily automatic processes of thought, induced by habit, closely resemble those which are instinctive. To the latter class belong the intuitive belief. To believe the information of our senses, as that the sun moves, is an instinct. Such also, is the expectation of like consequents from like antecedents, or cause and effect. Such, also, is the confident belief in the existence of outward things—the separation of the *ego* and *non-ego*. Such, also, the reference of the existence of things and of ourselves to an unseen Power. These, like other instincts, are variously susceptible of modification from subsequent experience. But in all these cases, the ideas have not been brought together by volitional efforts; they have an innate tendency to cohere. Indeed, they can hardly occur separately. The existence of the one necessarily involves that of the other. They, in fact, grow together. But the ideas which have only been casually clustered, or knit together by the workmanship of volition, may ultimately cohere as strongly. The force of habit will thus equal that of instinct; a faulty train of reasoning may acquire the strength of an original instinctive perception; an oft-told lie becomes a truth, and a superstition may be as hard to break asunder as the simplest and most primitive religion. Education thus vies with intuition—and habit becomes second nature.

These habits of thought, like habits of action, when once acquired are not easily discontinued. They are like grooves in which the mind has been accustomed to slide; if well contrived and fitted, and in a right direction, they are of incalculable value. If not, they are injurious, they prevent the mind from moving in better ordered and more truthward tracks. In the progress of life it becomes more and more difficult to alter our habits. The grooves have worn deeper and deeper, and the volition is less and less willing to trace or carve new ones. It is ridiculous, I should better say, it is mortifying to human nature to watch the awkward and futile efforts of a mind to throw off

* The whole subject of the automatic action of the brain in intellectual processes is treated with great ability and originality in Dr. Carpenter's Chapter on the Cerebrum and its Functions—("Principles of Human Physiology," fourth Edition). The reflex action of the brain with reference to motion had previously been expounded in a highly ingenious paper by Dr. Laycock, read before the British Association, September, 1844.

its long formed habits. Take it out of these prepossessions, and prejudices, and bigotries, and how uneasy and jolting are its movements! for such habits are ruts rather than grooves, which though they allow no rapid motion of the heavy carriage that follows them, yet cannot be escaped. In attempting to quarter the road the movements are made without confidence, and the wheels are perpetually threatening to slip into the old tracks with a crash that endangers the safety of the whole vehicle.

It would be interesting, but time will not allow us, to pass on to an investigation of habit in reference to the emotions and moral sentiments. But the same law will be found to hold good in that department as in those which we have so imperfectly treated.

I must conclude with a few words on the *final cause* of habit.—

As to habits of action, it is obvious that the great use they serve is the economy of time. What would man have accomplished by the end of his life had it been needful for him to attend to his movements in standing, walking, and using his hands and fingers? What progress would thought make, were speakers to be thinking of the sounds they utter, and to be consciously directing and adjusting their vocal apparatus? and where would be the literature of the world, were the mind compelled to pass from its sublime contemplations to the muscular actions which guide the movements of the pen? But the more we consider the subject, whether as to the development of those actions which characterize the species, or as to those acquired accomplishments and dexterities which range from the humblest handicrafts to the loftiest triumphs of the imaginative arts, the more we shall be struck by the gradually increasing subordination and subjugation of the mechanical processes to the more exalted faculties of the mind.

This view would at first, perhaps, make us inquire whether, as these volitional movements which we have been considering ultimately become automatic, it would not have enlarged the capacities of man, had they *begun* as instincts, just as some of them really are found in the lower animals, instead of going through so long a process of evolution and education? A foolish question, as every question must be, which proposes an arrangement of events different from what is obviously a part of the plan of God's universe. Take away the struggling striving will even from these corporeal actions; remove effort, resolution, the conscious initiation of action, perseverance, training, and education, and what is human life reduced to? Gigantic as man's powers become, he was not intended to spring from the earth in their full equipment. Survey him in his infancy, childhood, youth, adolescence, and manhood, and while you become convinced that his gradual acquirements bring him a multitude of enjoyments, as well as difficulties and disasters, you cannot but see that what is evolving in him bears a strict correlation to the powers, emotions, sentiments, and virtuous actions of those, who having arrived at the maturity of *their* powers, are to help *him*, to whom *he* is bound, as they to *him*, by ties which make the affinities of the human family infinitely transcend the transitory parental instincts and gregarious associations of the lower animals; for *they* live and grow up almost as they were born, devoid of progress, not one whit wiser or more skilful than the first pair that issued from Noah's ark, living for themselves only, or only under a blind impulse providing for another succession. But man having consciously and with pain and labour and peril, acquired his endowments, lives them over again by teaching them to his offspring; and apart from that happier existence to which he knows that he is destined in other worlds, feels that here too he has a kind of immortality; that as he has inherited knowledge, and virtue, and power, he too has to transmit them; that his life and its achievements have a mortal metempsychosis, a translation into the enlarging attributes and brightening destinies of his children, and of unborn generations, and in the production of works which, like Milton, he knows that posterity will not willingly let die, and in the elaboration of systems which, like Bacon, he bequeaths with his fame to the next ages; in this realizing anticipation of a posthumous renown, he survives his own death, passing by his living consciousness far beyond the narrow bounds affixed to his mere corporeal duration.

But while habit, as we have seen, is so useful in abridging labour, in economizing time, in preserving order, and method, and coherence in our thoughts, and in making the practice of virtue and religion easier to us, still it imposes upon us no inevitable compulsion. It is not the blind necessity of an instinct. It is our own fault if we are enslaved instead of being merely assisted by habit. Human agency ought to be able to assert its freedom in this as in every other department of thought and action. The habit should be like a steed, so well broken, that though the will may have thrown the reins on its neck, while otherwise occupied, it can in a moment gather them up, and come to a sudden halt.

Habit, we have seen at once, is the product and the sign of previous volition. And though in certain muscular actions belonging to the species, it closely resembles instinct, yet as to the thoughts and actions of individual men it is widely different. For as the will of every man has its own peculiar form and colour, making an important part of his individuality, so his habits will have their own character and freedom of growth. Those who are attached to him will regard with partiality the very habits which have grown out of his peculiarities. The singularities of his gestures, the eccentricities of his gait, carriage, and demeanour, the oddity of his featural expression, the tone of his voice, his ways and his whims, his fancies and his philosophies, his predilections and prejudices, the whole complexion of his life, and the whole colour of his conduct—his goings out and his comings in, his risings up and his lyings down,—all are valued, because they give us more vividly the express image of him who is endeared to us for his own individual sake.

But while there is the utmost latitude for the formation and the relinquishment of habit according to the will and humour of the individual, the habit which has grown out of a strong character will inevitably impress itself on others. A strong character does not depend on the intellect, but on the original strength of the will, and on the habits which it has originated. It is one whose actions have sprung out of its own individual will. The very limitation of the intellectual vision may strengthen the volition by the repetition of its objects and by the iteration of its correlative actions. This self-possessed, self-contained, self-originating power, is sure to affect others. It is a new centre of motion to those who are ever ready to derive their impulses from without, rather than from their own life.

Thus we see the two elements in social life antagonizing each other, and yet bringing out the most important results;—the potential freedom of every individual—and yet the unfelt compulsion of a passive imitation. And were any argument needful beyond what has been so often urged by moralists and divines as to the formation of habits which may become so powerful either for good or evil, in the *individual* character, it is to be found in the consideration that in our influence on others, we are responsible not only for what we directly do or directly teach, but also for that insensible operation of our characters which, in proportion as they are strengthened by habit, are in that same proportion sustained in their capability of impressing and moulding to their likeness, the wills, the affections, the thoughts, and the actions of our fellow men.

---

